# Optic Nerve Degeneration after Retinal Ischemia/Reperfusion in a Rodent Model

**DOI:** 10.3389/fncel.2017.00254

**Published:** 2017-08-22

**Authors:** Marina Renner, Gesa Stute, Mohammad Alzureiqi, Jacqueline Reinhard, Susanne Wiemann, Heiko Schmid, Andreas Faissner, H. Burkhard Dick, Stephanie C. Joachim

**Affiliations:** ^1^Experimental Eye Research, University Eye Hospital, Ruhr-University Bochum Bochum, Germany; ^2^Department of Cell Morphology and Molecular Neurobiology, Faculty of Biology and Biotechnology, Ruhr-University Bochum Bochum, Germany

**Keywords:** ischemia/reperfusion, retinal ischemia, optic nerve, microglia, macroglia, oligodendrocytes, neurofilament

## Abstract

Retinal ischemia is a common pathomechanism in many ocular disorders such as age-related macular degeneration (AMD), diabetic retinopathy, glaucoma or retinal vascular occlusion. Several studies demonstrated that ischemia/reperfusion (I/R) leads to morphological and functional changes of different retinal cell types. However, little is known about the ischemic effects on the optic nerve. The goal of this study was to evaluate these effects. Ischemia was induced by raising the intraocular pressure (IOP) in one eye of rats to 140 mmHg for 1 h followed by natural reperfusion. After 21 days, histological as well as quantitative real-time PCR (qRT-PCR) analyses of optic nerves were performed. Ischemic optic nerves showed an infiltration of cells and also degeneration with signs of demyelination. Furthermore, a migration and an activation of microglia could be observed histologically as well as on mRNA level. In regard to macroglia, a trend toward gliosis could be noted after ischemia induction by vimentin staining. Additionally, an up-regulation of *glial fibrillary acidic protein (GFAP)* mRNA was found in ischemic optic nerves. Counting of oligodendrocyte transcription factor 2 positive (Olig2^+^) cells revealed a decrease of oligodendrocytes in the ischemic group. Also, *myelin basic protein (MBP)* and *myelin oligodendrocyte glycoprotein (MOG)* mRNA expression was down-regulated after induction of I/R. On immunohistological level, a decrease of MOG was detectable in ischemic optic nerves as well. In addition, SMI-32 stained neurofilaments of longitudinal optic nerve sections showed a strong structural damage of the ischemic optic nerves in comparison to controls. Consequently, retinal ischemia impacts optic nerve degeneration. These findings could help to better understand the course of destruction in the optic nerve after an ischemic insult. Especially for therapeutic studies, the optic nerve is important because of its susceptibility to be damaged as a result to retinal ischemic injury and also its connecting function between the eye and the brain. So, future drug screenings should target not only the retina, but also the functionality and structure of the optic nerve. In the future, these results could lead to the development of new therapeutic strategies for treatment of ischemic injury.

## Introduction

Ischemia plays an important role in various retinal diseases, including age-related macular degeneration (AMD), diabetic retinopathy, glaucoma and retinal vein occlusion. These diseases are leading causes of blindness worldwide (Cai et al., 2015). Due to their complexity, the exact pathogenesis is still unclear. The retina has a high metabolic demand and reacts very sensitively to an impaired blood flow and thus an under-supply of nutrients (Kaur et al., 2008; Minhas et al., 2012). In the retinal ischemia/reperfusion (I/R) animal model such a circulatory disorder of the retina is induced by elevating the intraocular pressure (IOP) for a definite period of time. Because of the induced high IOP, retinal blood vessels are compressed, which in turn leads to an impaired blood flow. The following natural reperfusion also induces increased oxidative stress. Due to the renewed oxygen supply a higher concentration of the reactive oxygen species ensues, which triggers the formation of free radicals. These radicals attack the cell structure and proteins and cause additional massive tissue damage (Szabo et al., 1991; Belforte et al., 2011). Until now, several studies could demonstrate that I/R is followed by morphological and functional changes of different cell types of the retina (Dijk et al., 2004; Kaur et al., 2008; Belforte et al., 2011; Joachim et al., 2012; Minhas et al., 2012; Schmid et al., 2014). Retinal cell loss could be shown at an early point in time by several research groups (Lam et al., 1999; Zheng et al., 2004). Additionally, previous studies by our group could reveal that retinal ischemia also initiates a late loss and damage of different retinal cell types. A decline of retinal ganglion cells (RGCs) by apoptosis as well as less amacrine cells could be detected 21 days after ischemia. In addition, an increased number of inflammatory microglia was noted due to the strong tissue damage (Schmid et al., 2014). Regarding the optic nerve investigation, these immune cells are of particularly interest. Due to their ability to perform antigen presentation and elimination of cell debris by phagocytosis, invading microglia serves as indicators for early tissue damage and play moreover an essential role in initiation and resumption of de- and remyelination (Voss et al., 2012). Furthermore, a functional loss of the inner retinal cell layers could be measured in previous studies by full-field flash electroretinography 21 days after I/R induction (Schmid et al., 2014).

Because of the massive retinal damage, including the damage to the RGCs and the nerve fiber layer, it is assumed that the optic nerve is also impaired by the ischemic processes and is involved in the degeneration process. The unmyelinated axons of the RGCs bundle in the optic nerve head (ONH), which become myelinated in the retrolaminar and the orbital parts of the optic nerve, and continue to their targets representing the connection to the brain. Thus, the question arises, which consequences the I/R has on the optic nerve.

Some functional and structural changes in the optic nerve, following a transient ischemia, were already observed (Adachi et al., 1996; Grozdanic et al., 2003; Joachim et al., 2011). The group of Adachi et al. (1996) analyzed the optic nerve by light and electron microscopy 7 days after I/R for different periods of ischemia induction. They showed a degeneration of myelinated axons with disordered myelin sheaths even at 45 min of ischemia. At 60 min of ischemia, a distinct axonal damage in the form of swollen and collapsed axons and at 90 min of ischemia a fragmentation of myelin sheaths was revealed (Adachi et al., 1996). Also Joachim et al. (2011) could determine a significant axonal damage in optic nerve cross-sections as well as gliosis 2 and 4 weeks after 60 min of ocular ischemia. However, detailed morphological studies to evaluate the impact of retinal ischemia and the cell structures have not been performed yet.

The goal of this study was to investigate the effect of retinal ischemia induction on the optic nerve tissue. We wanted to find out, which cells and structures are specifically damaged on the different levels. Therefore, we have used immunohistological and molecular biological methods. For protein evaluation, several cell types such as micro-, macro- and oligodendroglia were analyzed using immunohistology. Additionally, quantitative real-time PCR (qRT-PCR) analyses were performed to evaluate the cellular markers on mRNA level. Attention was paid to the structural integrity as well as components of oligodendrocyte myelin sheaths of optic nerves of control and ischemic eyes.

## Materials and Methods

### Animals

Male Brown-Norway rats (7–8 weeks old; Charles River Laboratories, Sulzfeld, Germany) were used for this study. The study was approved by the animal care committee of North Rhine-Westphalia (Germany) and the experiments were carried out in accordance with the ARVO statement for the use of animals in ophthalmic and vision research. Rats were housed under environmentally controlled conditions (12-h light-dark cycle) with free access to chow and water.

### Induction of Ischemia/Reperfusion

Retinal I/R was induced as previously described (Schmid et al., 2014). Animals were anesthetized with a ketamine/xylazine/vetranquil cocktail (0.65/0.65/0.2 ml; 1.5 ml/kg body weight). One eye per animal was dilated using 5% tropicamide (40 μl/eye; Pharma Stulln, Stulln, Germany) and anesthetized topically with conjuncain (40 μl/eye; Bausch & Lomb, Berlin, Germany). Additionally, carprofen (2 ml/kg body weight; Pfizer, Berlin, Germany), a non-steroidal anti-inflammatory drug, was applied subcutaneously. IOP was raised to 140 mmHg for 60 min. This was done by elevating a saline reservoir containing 0.9% NaCl (Fresenius SE & Co. KGaA, Bad Homburg, Germany) and connected to a 27-gauge needle (Terumo Europe, Leuven, Belgium), which was placed into the anterior chamber of one eye. Retinal ischemia was confirmed by observing whitening of the retina and reperfusion was reassured by observing the returning blood flow with an ophthalmoscope (Mini 300; Heine Optotechnik, Herrsching, Germany). The other eye remained untreated and served as control (Lee et al., 2011). During the whole surgical intervention, the animals were kept on a heating pad to ensure a constant body temperature.

### Tissue Collection and Processing

Twenty-one days after I/R, optic nerve tissue was removed and processed for (immuno-) histology (*n* = 5/group) and qRT-PCR (*n* = 4/group). For (immuno-) histology, the optic nerves were fixed in 4% paraformaldehyde, incubated in 30% sucrose, embedded in optical cutting temperature medium (Tissue-Tek; Thermo Fisher Scientific, Cheshire, UK), and stored at −80°C (Shindler et al., 2006; Cho et al., 2011; Lee et al., 2011; Noristani et al., 2016). Longitudinal optic nerve sections, 4 μm thick, were prepared with a cryostat (Thermo Fisher Scientific, Walldorf, Germany) for further staining. For qRT-PCR analyses, the optic nerves were snap frozen in liquid nitrogen and stored at −80°C until RNA extraction.

### Histology

Longitudinal sections were stained with hematoxylin and eosin (H&E), luxol fast blue (LFB) and toluidine blue. H&E staining was realized to investigate structure alterations and cell infiltration. After the H&E staining, all slides were dehydrated in ethanol following incubation in xylene before being mounted with Eukitt (O-Kindler GmbH & Co, Freiburg, Germany). LFB as well as toluidine blue staining were used to examine demyelination. In order to stain myelin selectively, optic nerve sections were stained with LFB without cresyl violet. Differentiation in a solution of lithium carbonate followed. Subsequently, a dehydration in ethanol and an incubation in xylene followed, before the slides were mounted with Eukitt. For toluidine blue staining, sections were immersed in a solution of toluidine blue and 1% sodium chloride followed by washing in aqua dest. After drying, the slides were mounted with Eukitt. Of each staining, three areas per optic nerve section were documented with a microscope equipped with a CCD camera (Axio Imager M1; Carl Zeiss Microscopy) as previously described (Noristani et al., 2016; Reinehr et al., 2016a). One photo was taken of the proximal part, just behind the ONH, one of the middle and one of the distal part, right in front of the chiasma. In total, six sections per optic nerve were stained and photographed.

Cell infiltration in optic nerve sections stained with H&E was evaluated by using a scoring system ranging from 0 to 4 by a masked observer: 0 = no infiltration, 1 = mild cellular infiltration of the optic nerve or optic nerve sheath, 2 = moderate infiltration, 3 = severe infiltration, and 4 = massive infiltration of the optic nerve parenchyma and nodule infiltration (Shindler et al., 2006; Horstmann et al., 2013). The average score for each optic nerve was used for statistical analysis.

The grade of demyelination was monitored in optic nerve sections stained with LFB. Therefore, staining was scored from 0 to 2 and categorized as follows: 0 = no demyelination, 1 = moderate demyelination, and 2 = severe demyelination up to dissolution of the tissue. The average score for each optic nerve was used for later statistical evaluation (Calida et al., 2001; Horstmann et al., 2013; Liu et al., 2014).

### Immunohistology of Optic Nerve Sections

Longitudinal optic nerve sections (*n* = 5/group) were also prepared for immunohistochemistry. After drying and rehydration in PBS, sections were blocked in 10%–20% appropriate serum with 1% BSA in 0.1% or 0.2% Triton X-100 in PBS. Six longitudinal sections per optic nerve were used for each staining. The whole microglia population was stained with anti-Iba1 (1:400; Wako Chemicals, Neuss, Germany), activated microglia and macrophages were labeled with anti-ED1 (1:200; Millipore, Darmstadt, Germany). Corresponding secondary antibodies were used (Alexa 488, 1:500; Alexa 555, 1:500; both Invitrogen, Darmstadt, Germany). To investigate macroglial cells, the anti-glial fibrillary acidic protein (GFAP; 1:500; Millipore), specific for astrocytes, and vimentin (1:500; Sigma-Aldrich, Darmstadt, Germany) was used. The secondary antibody for GFAP was conjugated to Cy3 (1:500; Millipore). The staining for vimentin was followed by Alexa 555 (1:500; Abcam, Cambridge, UK) as secondary antibody. The myelin basic protein (MBP) was stained with anti-MBP (1:100; Millipore). The secondary antibody was conjugated to Cy3 (1:500; Millipore). The oligodendrocyte transcription factor 2 (Olig2) was used to identify oligodendrocytes (1:500; Sigma Aldrich), followed by the secondary antibody Alexa 488 (1:500; Invitrogen). With anti-myelin oligodendrocyte glycoprotein (MOG; 1:60; R&D Systems, Minneapolis, MN, USA) the myelin glycoprotein of oligodendrocytes was detected. As secondary antibody Alexa 488 (1:500; Invitrogen) was used. Neurofilament proteins were labeled with anti-SMI-32 (1:6000; Convance) and Alexa 488 (1:400; Invitrogen) as secondary antibody. As a nuclear stain 4′,6-Diamidin-2-phenylindol (DAPI; Serva Electrophoresis, Heidelberg, Germany) was used. Negative controls were performed by applying only the secondary antibody. Three pictures per optic nerve section were taken. One photo was taken of the proximal part, just behind the ONH, one of the middle and one of the distal part, right in front of the chiasma using a fluorescence microscope (Axio Imager M2; Carl Zeiss Microscopy) as previously described (Noristani et al., 2016; Reinehr et al., 2016a). All digitalized images were transferred to Corel Paint Shop Photo Pro (V 13; Corel Corp., Fremont, CA, USA), masked and excerpts of a defined area were cut out. Masking was done with the software “Ant Renamer”. According to the random principle, an eight-digit number was given to each image.

Evaluation was carried out under masked conditions with ImageJ software (V 1.44p; NIH, Bethesda, MD, USA). The Iba1^+^, ED1^+^ and Olig2^+^ cells were counted. In regard to ED1, the co-localization with Iba1 was evaluated.

For analysis of vimentin, MBP and MOG staining, the images were transferred to ImageJ and transformed into gray scale. After subtraction of the background (vimentin: 15 pixels; MBP: 20 pixels; MOG: 13 pixels), the lower and upper thresholds were set (vimentin: lower threshold: 6.4, upper threshold: 90; MBP: lower threshold: 18.02, upper threshold: 125; MOG: lower threshold: 8.8, upper threshold: 125). Background subtraction and lower and upper threshold represent mean values of both groups. For each picture, the percentage of the vimentin^+^, MBP^+^ and MOG^+^ labeled area was measured using an ImageJ macro (Schmid et al., 2014; Casola et al., 2015).

Staining of SMI-32 was analyzed using an established scoring system ranging from 0 to 2. The score of the staining was graded as follows: 0 = intact structure, no retraction bulbs, 1 = shortened axons, some retraction bulbs, and 2 = loss of structural integrity and plenty of retraction bulbs and holes (Noristani et al., 2016). For statistical evaluation, the average score for each optic nerve was used.

### Quantitative Real-Time PCR Analysis of Optic Nerve Tissue

A change in protein level is detectable before or simultaneously on mRNA level. In order to also analyze the mRNA level of the various cellular and structural markers, qRT-PCR analyses were performed. Total RNA from optic nerve tissue (*n* = 4/group) was extracted and purified according to the manufacturer’s instructions using the ReliaPrep™ RNA Tissue Miniprep System (Promega, Madison, WI, USA). Before lysis, optic nerves were snap frozen in liquid nitrogen. RNA concentration and purity was assessed by spectrophotometry (BioSpectrometer; Eppendorf, Hamburg, Germany). To obtain cDNA, 1 μg of total RNA was reverse-transcripted by means of a cDNA-synthesis kit and random hexamer primers (Thermo Fisher Scientific, Waltham, MA, USA). qRT-PCR experiments were performed with SYBR Green I in a Light Cycler 96 (Roche Applied Science, Mannheim, Germany). Primer efficiencies of each primer set were calculated based on a dilution series of 5–125 ng cDNA (Reinehr et al., 2016b). For normalization and relative quantification, Ct values of the house-keeping gene *cyclophilin* were taken into account (Table 1).

**Table 1:** List of primer pairs used for analyses of micro- and macroglia, neurofilament as well as oligodendrocyte mRNA expression in control and ischemic optic nerves by quantitative real-time PCR (qRT-PCR).

Gene	Primer sequence	Amplicon size	Primer efficiency
*Cyclophilin-F* *Cyclophilin-R*	tgctggaccaaacacaaatg cttcccaaagaccacatgct	88 bp	0.939
*Iba1-F* *Iba1-R*	ctccgaggagacgttcagtt tttttctcctcatacatcagaatcatcagaat	96 bp	0.844
*CD68-F* *CD68-R*	tctgaccttgctggtactgc gaagagtggcagcctttttg	74 bp	0.873
*GFAP-F* *GFAP-R*	tttctccaacctccagatcc gaggtggccttctgacacag	64 bp	0.878
*MBP-F* *MBP-R*	ggcacgctttccaaaatct ccatgggagatccagagc	61 bp	0.909
*MOG-F* *MOG-R*	gcaggtctctgtaggccttg gcacggagttttcctctcag	63 bp	0.892
*Olig2-F* *Olig2-R*	gcgcgaaactacatcctga cgtaaatctcgctcaccagtc	70 bp	0.773
*Vimentin-F* *Vimentin-R*	ttcttccctgaacctgagaga ggagtgggtgtcaaccagag	61 bp	0.955

*For relative quantification of mRNA levels the house-keeping gene *cyclophilin* was used. The primer sequence, the predicted amplicon size, primer efficiency and the reference are indicated. Abbreviations: bp, base pairs, F, forward, R, reverse*.

### Statistics

Histological data are presented as mean ± SEM and qRT-PCR data as median ± quartile + minimum + maximum. Regarding histology, groups were compared using Student’s *t*-test (Statistica V13.0; Dell, Tulsa, OK, USA). For statistical evaluation of relative expression variations in qRT-PCR analyses, data were analyzed by REST^©^ software (QIAGEN GmbH, Hilden, Germany) using a pairwise fixed reallocation and randomization test. *P*-values below 0.05 were considered statistically significant.

## Results

**Figure 1:**
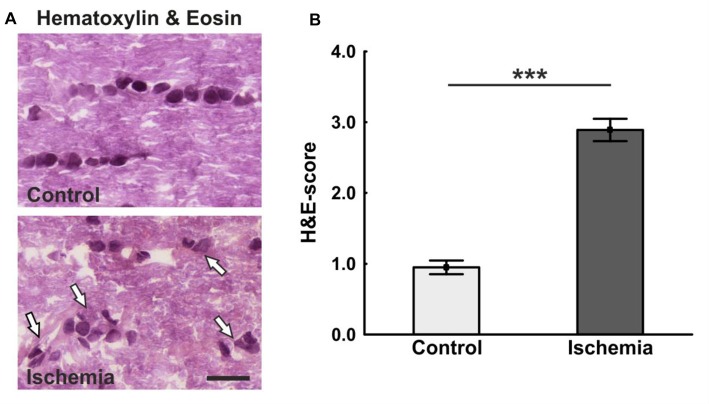
**(A)** Longitudinal optic nerve sections were stained with hematoxylin & eosin (H&E). Cell cluster and a resolution of the tissue could be noted after ischemia/reperfusion (I/R). In the control group, the cells were arranged in series. **(B)** The cell infiltration was determined using a scoring system ranging from 0 (no infiltration) to 4 (massive infiltration). Significantly more cells infiltrated the optic nerves of ischemic animals. Arrows = cluster of cells. Scale bar: 20 μm. ***p < 0.001.

**Figure 2:**
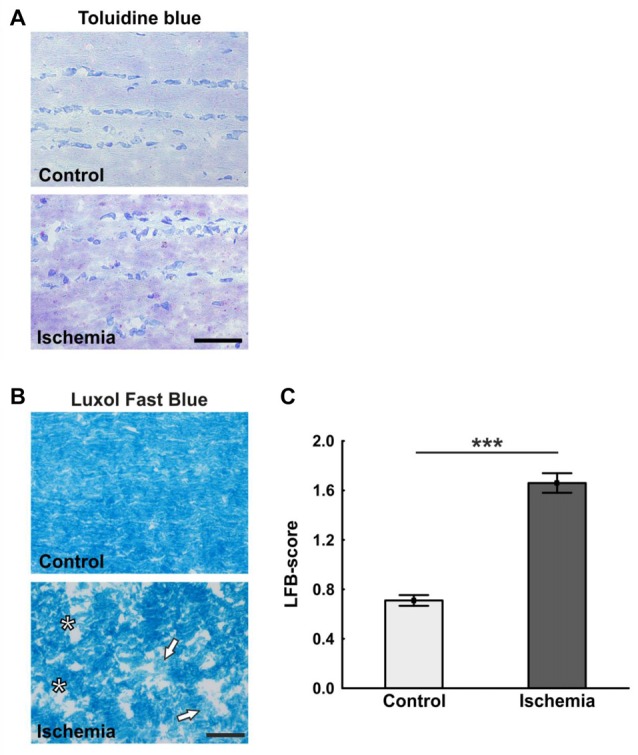
**(A)** Representative pictures of the toluidine blue staining. While in the control group a uniform coloring was present, various light areas were visible in the ischemia group. **(B)** Staining of longitudinal optic nerve sections with luxol fast blue (LFB). Comparable to toluidine blue staining, large bright areas were seen in the ischemic optic nerves. In contrast to this, the control optic nerves were uniformly colored. **(C)** The loss of myelin was valued based on a scoring system. Many areas of degeneration with subsequent loss of myelin and a dissolution of the tissue could be observed 21 days after ischemia induction. Arrows = demyelination area, asterisks = structure resolution. Scale bar: toluidine blue 50 μm, LFB 20 μm. ***p < 0.001.

The ischemic longitudinal optic nerve sections stained with H&E showed a destroyed structure of the optic nerve tissue (Figure 1A). Significantly more infiltrating cells in the form of cell clusters were observed 21 days after ischemia induction (2.9 ± 0.2) compared to controls (1.0 ± 0.1; *p* < 0.001; Figure 1B). There, the cells were arranged in a row via toluidine blue staining, bright areas could be recognized in the ischemic optic nerves, which indicate a demyelination. In comparison, optic nerves of the control group showed a uniform toluidine blue staining (Figure 2A). Staining of the myelin sheaths with LFB showed degeneration with subsequent loss of myelin sheaths after ischemia. Bright large areas right up to tissue dissolution could be identified in the ischemic optic nerves. In comparison, the control group showed a consistent uniform coloration (0.7 ± 0.04; Figure 2B). Statistical analysis revealed a significant damage with signs of demyelination in the ischemic group (1.7 ± 0.1; *p* < 0.001; Figure 2C).

**Figure 3:**
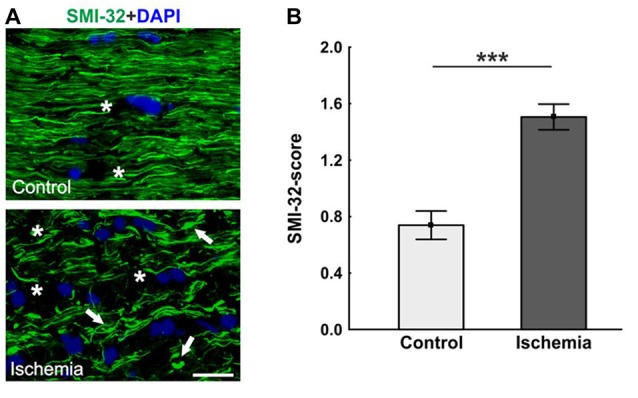
**(A)** Optic nerve sections labeled with SMI-32 for neurofilament (green) and 4′,6-Diamidin-2-phenylindol (DAPI) for cell nuclei (blue). In comparison to the control group, many retraction bulbs, short axons and holes could be noted in ischemic optic nerve tissue. **(B)** Scoring of the SMI-32 stained sections revealed a significant structural distortion of the optic nerves of the ischemic group. Arrows = retraction bulbs, asterisks = holes. Scale bar: 20 μm. ***p < 0.001.

Neurofilament proteins were marked using SMI-32. The control optic nerves had long nerve fibers, which were arranged in parallel, while sections of ischemic optic nerves had a lot of retraction bulbs and short axons (Figure 3A). Statistical analysis of the scoring revealed a significant axonal damage of the optic nerves of the ischemic group (1.5 ± 0.1; *p* < 0.001) in comparison to the control group (0.7 ± 0.1; Figure 3B).

**Figure 4:**
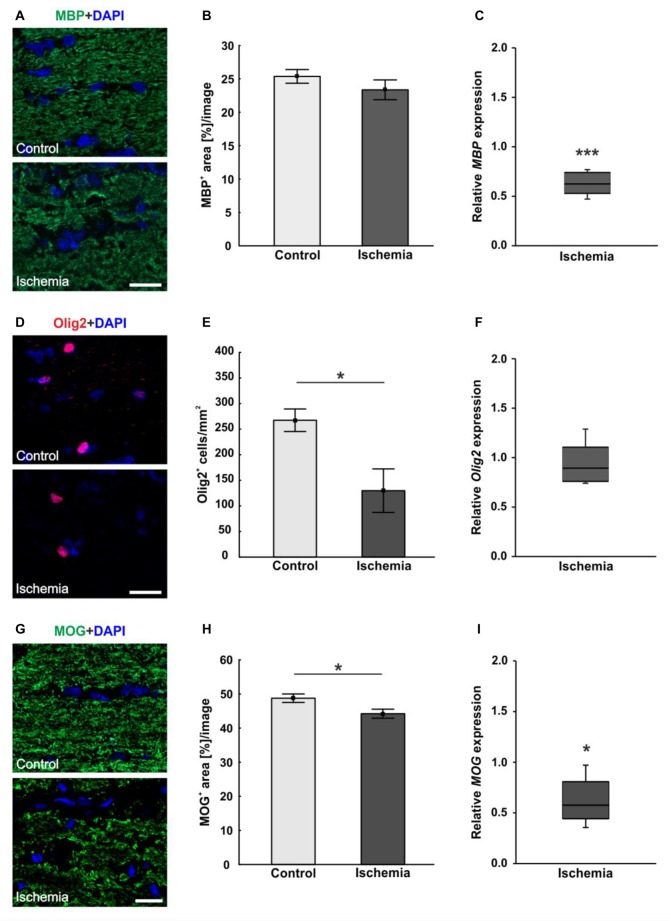
**(A)** Myelin basic protein (MBP, green), and DAPI (cell nuclei; blue) staining of optic nerve sections. No differences in immunoreactivity of MBP could be seen, but a destruction of the ischemic tissue could be observed. **(B)** Statistical analysis showed no differences in MBP^+^ area between both groups. **(C)** Otherwise, a significant decrease of *MBP* mRNA was noted in optic nerves of ischemic eyes in relation to controls. **(D)** Oligodendrocyte transcription factor 2 (Olig2) was used to stain oligodendrocytes (maturing oligodendrocyte precursor cells (OPCs), red), DAPI was used for cell nuclei (blue). Regarding the Olig2^+^ cells, fewer cells were observed in the ischemic group. **(E)** Counting of Olig2^+^ cells revealed a significantly decreased cell number in the ischemic group compared to controls. **(F)** On mRNA level, no differences could be observed between both groups. **(G)** With myelin oligodendrocyte glycoprotein (MOG) the myelin glycoprotein of oligodendrocytes was marked (green). DAPI was used for cell nuclei (blue). A lower MOG signal and a resolution of the tissue could be shown in ischemic optic nerves. **(H)** Statistical analyses revealed a significant reduction in the MOG^+^ area after ischemia induction. **(I)** Also via quantitative real-time PCR (qRT-PCR), a significant decrease of *MOG* mRNA expression was observed in ischemic optic nerves in comparison to controls. Scale bar: 20 μm. *p < 0.05, ***p < 0.001.

An antibody specific for MBP was used to visualize the basic protein of myelin, a major constituent of oligodendrocyte myelin sheaths. No differences in MBP staining area could be seen, whereas a structural distortion of the tissue of ischemic optic nerves could be noted (Figure 4A). Statistical analyses of the immunohistological staining confirmed this impression. No differences could be detected between both groups in regard to MBP^+^ area (ischemia: 23.4 ± 1.5% area/image; control: 25.4 ± 1.0% area/image; *p* = 0.3; Figure 4B). In order to evaluate the mRNA level, *MBP* expression was analyzed via qRT-PCR. These results showed a significant down-regulation of relative *MBP* expression in ischemic optic nerves compared to control ones (0.624-fold expression; *p* < 0.001; Figure 4C).

Oligodendrocytes were detected using anti-Olig2. The oligodendrocyte lineage transcription factor marks maturing oligodendrocyte precursor cells (OPCs) and is required for the oligodendrocyte specification and differentiation (Marsters et al., 2016). Less Olig2^+^ cells were perceived in optic nerves of the ischemic group (Figure 4D). The statistical evaluation reflected this observation. The optic nerves of the ischemic eyes showed a significant Olig2^+^ cell loss (129.8 ± 42.5 cells/mm^2^) when compared to the control group (267.5 ± 22.0 cells/mm^2^; *p* = 0.02; Figure 4E). Also, the *Olig2* expression was analyzed via qRT-PCR. However, no differences in the expression of *Olig2* could be detected between both groups (0.891-fold expression; *p* = 0.39; Figure 4F).

The myelin glycoprotein of oligodendrocytes was stained with anti-MOG and the positive area was evaluated. The protein is located on the surface of myelinating oligodendrocytes and external lamellae of myelin sheaths (Pham-Dinh et al., 1993). A resolution of the structure as well as a disorganization and less immunoreactivity were detectable in ischemic optic nerves (Figure 4G). Evaluation of the area percent confirmed this impression. A significantly smaller MOG^+^ area could be observed in ischemic optic nerves (44.2 ± 1.3% area/image) compared to the control group (48.8 ± 1.2% area/image; *p* = 0.03; Figure 4H). Also on mRNA level, a significant down-regulation of *MOG* expression was measured after I/R (0.574-fold expression; *p* = 0.012; Figure 4I).

**Figure 5:**
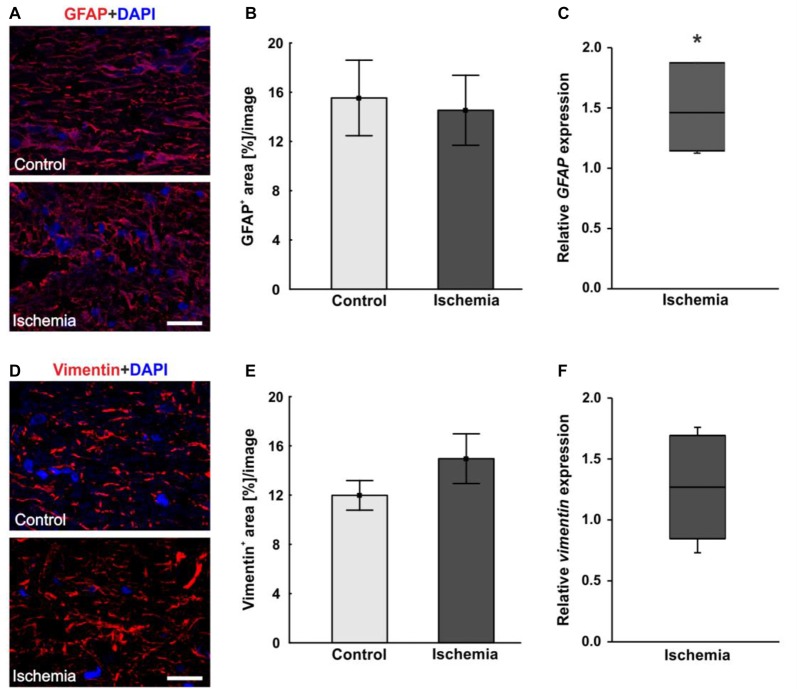
**(A)** Macroglial staining of optic nerve sections with glial fibrillary acidic protein (GFAP; red) and DAPI for cell nuclei (blue). An unstructured GFAP immunoreactivity could be observed after I/R. **(B)** Evaluation of GFAP stained sections revealed no differences in GFAP^+^ area between the optic nerves of both groups. **(C)** On mRNA level, a significant up-regulation in *GFAP* expression could be shown in the ischemia group in relation to controls. **(D)** Additional staining of macroglia with vimentin (red). DAPI was used to label cell nuclei (blue). **(E)** No differences could be noted in vimentin^+^ area between both groups, but an increasing trend could be observed in the ischemic group. **(F)** Compared to controls, also on mRNA level, no differences in *vimentin* expression were measured in ischemic optic nerves. Scale bar: 20 μm. *p < 0.05.

In order to visualize macroglia, antibodies against GFAP and vimentin were used. Both intermediate filaments are mainly expressed by astrocytes. In the ischemic optic nerves, a structural resolution and a disorganized and unstructured immunoreactivity of GFAP^+^ and vimentin^+^ area could be observed (Figures 5A,D). Evaluation of the GFAP^+^ area revealed no differences between optic nerves of the ischemic group (14.5 ± 2.8% area/image) and control ones (15.5 ± 3.1% area/image; *p* = 0.8; Figure 5B). In regard to vimentin signal, no differences in vimentin^+^ area could be noted between both groups, although an increasing trend could be observed in the ischemic group (ischemia: 15.0 ± 2.0% area/image; control: 12.0 ± 1.2% area/image; *p* = 0.2; Figure 5E). In addition, the relative *GFAP* and *vimentin* expression was analyzed via qRT-PCR. A significant up-regulation of *GFAP* mRNA could be measured in the ischemic group (1.458-fold expression; *p* = 0.014) when compared to controls (Figure 5C). Regarding *vimentin* mRNA expression, no differences were detectable after ischemia induction on mRNA level (1.268-fold expression; *p* = 0.222; Figure 5F).

**Figure 6:**
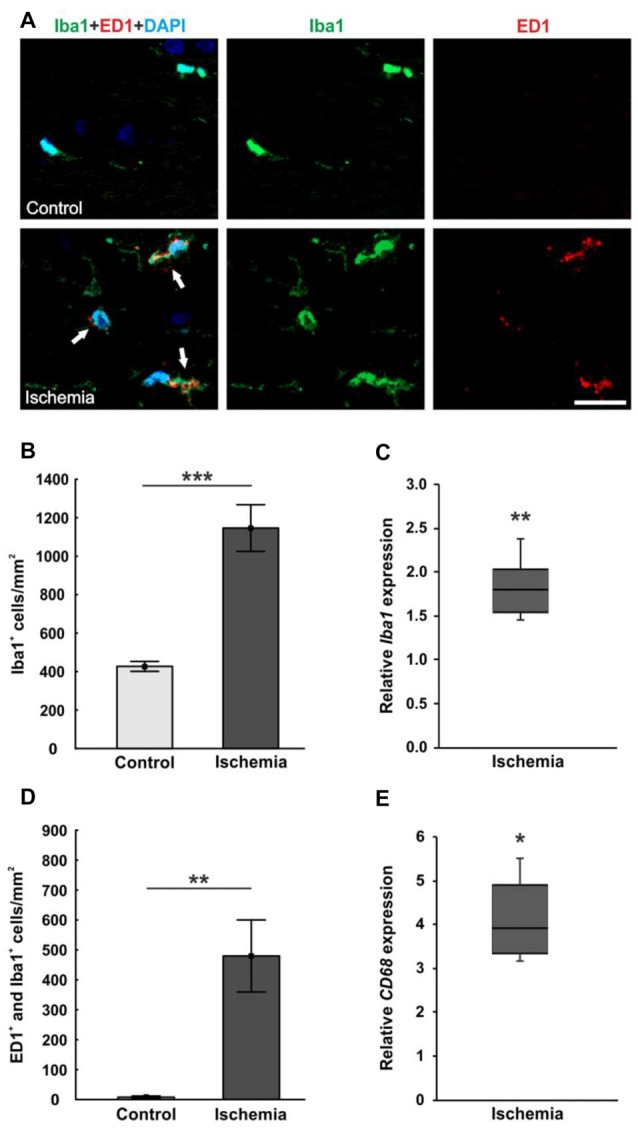
**(A)** Optic nerve sections were stained with Iba1 (microglia; green), ED1 (active microglia; red) and DAPI (cell nuclei; blue). Much more microglia as well as activated ones were present in the tissue of ischemic optic nerves. **(B)** Significantly more Iba1^+^ microglia were detected in the optic nerves of the ischemic group. **(C)** Also, via qRT-PCR, a significant increase of *Iba1* mRNA expression was observed in ischemic optic nerves in comparison to controls. **(D)** A significant activation of microglia could be revealed in the ischemic group. Significantly more ED1^+^ and Iba1^+^ microglia were counted. **(E)** Equally, *CD68* mRNA (activated microglia) expression was significantly up-regulated in ischemic optic nerves in relation to controls. Arrows = co-localization of ED1^+^ and Iba1^+^ cells. Scale bar: 20 μm. *p < 0.05, **p < 0.01, ***p < 0.001.

The microglia population was detected with the specific anti-Iba1 antibody, while an anti-ED1 antibody was used to mark activated microglia. A lot more microglia were noted in ischemic optic nerves, many of them were activated (Figure 6A). Cell counts confirmed this observation. The number of Iba1^+^ microglia was significantly higher in optic nerves of ischemic eyes (1146.3 ± 121.2 cells/mm^2^; *p* < 0.001) compared to controls (427.2 ± 25.8 cells/mm^2^; Figure 6B). Also, significantly more activated microglia could be revealed in the ischemic group (479.8 ± 120.4 cells/mm^2^) in relation to the control group (7.9 ± 3.8 cells/mm^2^; *p* = 0.004; Figure 6D). In accordance with the immunohistological data, a significant higher *Iba1* mRNA expression was detected in ischemic optic nerves (1.803-fold expression; *p* = 0.004) compared to controls (Figure 6C). Equally, *CD68* mRNA (activated microglia) expression was significantly increased in the ischemic group (3.918-fold expression; *p* = 0.017) in relation to controls (Figure 6E).

## Discussion

Ischemia leads to severe damage of the retina. Various cell types from the inner to the outer layers are affected due to the disturbance of the blood supply. Loss of function and morphological changes and damage were described. Specifically, loss of RGCs and amacrine cells is documented (Sellés-Navarro et al., 1996; Lafuente et al., 2002; Dijk and Kamphuis, 2004; Schmid et al., 2014). Nevertheless, the extent of an ischemic injury on the optic nerve is not very well known. In order to better understand the effects and impact of a retinal ischemic injury on the optic nerve tissue, we investigated optic nerves via immunohistology and qRT-PCR. Especially, with regard to the ischemia induced RGC loss and degeneration of their axons, it has to be assumed that the optic nerve also suffers a damage. Our results confirm this assumption and demonstrate that besides the retinal damage, the impact extends to the optic nerve. We could detect neuronal degeneration, a tissue dissolution and structural distortion as well as increased microglia activation. Additionally, the astrocytes were up-regulated on mRNA level, a decrease of *MBP* and* MOG* mRNA, both components of oligodendrocyte myelin sheats, and fewer oligodendrocytes were noted.

### Structural Degeneration of the Optic Nerve

One component of the axon is the neurofilament, which can be detected by SMI-32. The neurofilament is important for the structural integrity of axons. We could show a structural distortion of the neurofilament 21 days after I/R. Also, in an experimental autoimmune glaucoma model, a neurofilament dissolution could be demonstrated at a late point in time, 28 days after immunization (Noristani et al., 2016). Ebneter et al. (2010) observed subtle neurofilament abnormalities in the ONH and the optic nerve already 3 days after inducing ocular hypertension via laser photocoagulation in an experimental glaucoma model. Seven days after induction of experimental glaucoma, they noticed numerous axons with SMI-32 abnormalities (Ebneter et al., 2010). Additionally, the group of Song et al. (2003) could show a visible diminution in neurofilament immunostaining 3 and 7 days after ischemia in a mouse model. Via diffusion tensor imaging (DTI), they could also verify axonal loss at this points in time, confirming their immunostaining observations (Song et al., 2003). Thus, the neurofilament seems to react very sensitively to ischemic processes.

Optic nerve axons are surrounded by myelin sheaths, which electrically isolate them. Myelin also protects the neurofilaments from inflammatory processes. A destruction of the axonal myelin sheaths leads to a disturbed signal transduction and thus to visual disorders. With the LFB staining, we could demonstrate that an ischemic retinal injury results in a significant degeneration of the axons. The observed whitening of the optic nerve tissue indicates a loss of the myelin sheaths. Significant changes of the myelin structure were also detected in the experimental autoimmune glaucoma model after 28 days (Noristani et al., 2016). Conveniently, we also noted a down-regulation of the expression level of the protein *MBP*, which is a major constituent of the myelin. In contrast, there was no difference on protein level. In addition, we have considered another component of myelin sheets, namely the protein MOG. Here, we could detect a significant reduction in both, protein and mRNA level. Due to this, it can be supposed that at the time of investigation, demyelination processes were started and are still ongoing. Possibly, the point in time was still too early to detect a structural extinction of the main myelin component MBP histologically. This assumption is supported by the detected significant loss of oligodendrocytes, which correlated with the severity of demyelination noted previously via LFB staining. Oligodendrocytes are assigned to the macroglia and accept the responsibility for myelination and metabolic support of the axon (Meyer et al., 2001; Lappe-Siefke et al., 2003). Thus, MBP is typically expressed in mature oligodendrocytes (Stock et al., 2010). MOG is localized on the surface of myelinating oligodendrocytes as well. Due to the degeneration of the oligodendrocytes after ischemia induction, the expression of *MBP* and* MOG* mRNA is down-regulated. After spinal cord injury, activated Olig2^+^ oligodendrocyte progenitor cells mediate remyelination. Thus, a migration, proliferation and differentiation of the progenitors near the injury site is necessary for oligodendrocyte myelination (Thomas et al., 2014). Since we marked mature oligodendrocytes as well as OPCs via Olig2, it can be supposed that a loss of both oligodendrocyte cell types occured. Thus, a remyelination could no longer be initiated. This would support our assumption of a progressive demyelination process in the optic nerve after ischemic injury. Other studies that have worked with a mouse model of anterior ischemic optic neuropathy (rodent AION), indicate a rapid dysfunctionality of oligodendrocytes after ischemia. In their mouse model the researchers could observe an oligodendrocyte loss via apoptosis in the anterior optic nerve within 2 days after rodent AION induction (Goldenberg-Cohen et al., 2005). However, the protective effect of myelin is no longer present, which makes the neurofilaments even more vulnerable. The structural distortion, noted via SMI-32, and the measured decrease of the myelin constituents, underpins this hypothesis.

Regarding the temporal course of the destruction, there are two options. The high IOP, which is induced, could be exerting pressure on the retina and the ONH. This direct mechanical damage to the ONH could then lead to a simultaneous degeneration of RGCs and axons. The other possibility is, that the optic nerve degeneration takes place delayed. After a previous RGC loss in the retina, the neurofilament and then the myelin degeneration might occur (Wallerian degeneration). This hypothesis is supported by the work of Song et al. (2003). Via DTI, they could show axonal and myelin degeneration at an early point in time after transient retinal ischemia. Also in an experimental acute glaucoma model in the owl monkey, signs of optic nerve degeneration were already observed a few days after glaucoma induction (Zimmerman et al., 1967). However, to clarify this, further studies at earlier points in time are necessary, where retinal as well as optic nerve tissue should be investigated at the same point in time to verify the site of the first damage. With regard to the mechanical aspect, it might be interesting to perform cerebral ischemia via artery occlusion and to investigate the optic nerves subsequently. In this case, no pressure would be exerted on the ONH. It could be then assumed that the damage is directly caused by ischemia.

### Macroglia Response on Ischemia Induction

The population of immune cells, which are located in the retina and optic nerve, are glia cells. They have the ability to react and become activated immediately in the case of damage. A subdivision is made into microglia and macroglia (astrocytes and oligodendrocytes; Tezel and Wax, 2007; de Hoz et al., 2016). Astrocytes embody the most common and morphologically heterogeneous neuroglia cell (de Hoz et al., 2016). They participate in information processing, neuronal circuits, and maintenance of synaptic integrity (Barateiro et al., 2016; de Hoz et al., 2016; Pekny et al., 2016). However, their main task is to control, protect, and support neuronal function (Pekny et al., 2014). It is known that ischemic damage, neurodegeneration, neuroinflammation, or trauma causes a process called reactive gliosis. This includes the up-regulation of cytoskeletal components, such as GFAP and vimentin, which are expressed by astrocytes (Ramírez et al., 2001; Peng et al., 2014). A reactive astrogliosis, including an increased GFAP expression, has been reported in different retinal pathologies and animal models of glaucoma (Hernandez et al., 2008; Gallego et al., 2012). We could also verify an increased expression of *GFAP* mRNA via qRT-PCR due to the ischemic damage. Immunohistologically, there was an increasing trend towards a higher vimentin expression. Regarding GFAP, we identified a structural disorganization and destruction of the astrocytes. Thus, in our study, the state of pathological gliosis was already transcended. Cho et al. (2011) observed gliosis of astrocytes in the ONH 2, 5 and 7 days after acute high IOP I/R. Additionally, they also noted a decrease of GFAP immunoreactivity 1 week after injury (Cho et al., 2011). It is assumed that glial cell activation represents the earliest response to CNS injury. Thus, the initial phase of astrocyte activation is considered a compensatory response to preserve the neuronal function (Cho et al., 2011). Possibly, the point in time, on which we investigated the optic nerves, was too late for this observation. After 21 days, ischemia induction appears to lead to the destruction of the astroglia structure. Thus, these cells could no longer exercise their protective function. The optic nerve tissue is vulnerable and could be attacked, e.g., by oxidative stress. This in turn could lead to further damage of the tissue and would match the previous data regarding the degeneration of the neurofilament and the myelin.

### Microglia Activity after Ischemic Injury

Microglia play an important role in the defense mechanism of the immune system with different functions, including the phagocytosis of foreign bodies and cell fragments. In case of a present damage, an inflammatory event or cellular stress the microglia are active and migrate to the location of the event. In addition to ischemia, this has also been described for neuronal injuries, retinopathies and photoreceptor degeneration (Kornek et al., 2000; Langmann, 2007; Horstmann et al., 2013). As a result of the ischemia induction in our study, we could also detect an increased number of microglia as well as more activated ones. In a multiple sclerosis model it has been described that micro- and macroglial cells are recruited in order to remove damaged myelin in the brain during the demyelination processes (Neumann et al., 2009; Voss et al., 2012). Since we have also seen a strong demyelination after I/R, this could be a possible reason for the increased activation of microglia in our study. Possibly, it can also explain the cell clusters we noted via the H&E staining. Also in an acute high IOP I/R model, an increased number of cells, which were interpreted as glial cells, were observed in the ONH 2 and 5 days after ischemia induction. After 1 week, the H&E staining showed a decrease of these cells (Cho et al., 2011). Otherwise, an increased number and activation of microglia in the optic nerve was documented in an experimental glaucoma model of ocular hypertension after 2 and again at 6 weeks. There, ED1+ microglia displayed an activated morphology that coincided with the severity of the optic nerve damage (Ebneter et al., 2010). The research group assumed that later time points of axonal injury will include morphologically visible axonal breakdown, myelin disruption as well as phagocytic system activation. Thus, the extent of microglia activation reflect in the severity of the injury (Ebneter et al., 2010). It is still discussed whether microglia activation has a protective or harmful effect. A theory implies that in an overactivated and dysregulated state, microglia aggravate an already existing damage by promoting a second disease course (Cardona et al., 2006; Block et al., 2007). This could explain, why we observed such a strong microglia activation in the ischemic optic nerves at this late point in time. A previous study of our research group, in which we could note a significant microglial activation in the retina at the same point in time, does also underline this assumption (Schmid et al., 2014).

## Conclusion

Our data show a marked cellular as well as structural damage and degeneration of the optic nerve tissue at a late point in time. Thus, optic nerve degeneration plays an important role in retinal I/R. However, it still needs to be clarified if the ischemic damage overlaps from the retina to the optic nerve or whether a second initial damage is set in the ONH by ischemia induction. I/R results in the loss of RGCs and their axons. This could lead to optic nerve degeneration with a subsequent loss of myelin. We suppose that due to this injury of the optic nerve, micro- and macroglial activation takes place. Due to the strength of the damage it should be assumed that the signal transduction to the brain is impaired. We conclude, that it is not sufficient to develop new treatment strategies for the retina alone. Our findings suggest, that the optic nerve also needs to be protected against ischemic injury. If the optic nerve is damaged or destroyed, no signal transduction to the brain would take place, even if retinal cells, especially RGCs and their axons, could be protected. Therefore, neuroprotective effects of potential therapies should be simultaneously investigated in the retina and the optic nerve structure.

## Author Contributions

MR performed experiments, analyzed data and wrote the manuscript. MA performed experiments and analyzed data. GS, SW and HS performed experiments. JR analyzed data and revised the manuscript. SCJ designed the study. AF, HBD and SCJ revised the manuscript. All authors read and approved the final manuscript.

## Conflict of Interest Statement

The authors declare that the research was conducted in the absence of any commercial or financial relationships that could be construed as a potential conflict of interest.
